# Four very basic ways to think about policy in implementation science

**DOI:** 10.1186/s43058-023-00497-1

**Published:** 2023-09-12

**Authors:** Jonathan Purtle, Corrina Moucheraud, Lawrence H. Yang, Donna Shelley

**Affiliations:** 1 Department of Public Health Policy & Management, Global Center for Implementation Science, New York University School of Global Public Health, 708 Broadway, New York, NY 10003 USA; 2 Department of Social and Behavioral Sciences, Global Center for Implementation Science, New York University School of Global Public Health, Global Mental Health and Stigma Program, 708 Broadway, New York, NY 10003 USA

**Keywords:** Policy, Outer-setting, Implementation strategies, Dissemination strategies, Education

## Abstract

**Background:**

Policy is receiving increasing attention in the field of implementation science. However, there remains a lack of clear, concise guidance about how policy can be conceptualized in implementation science research. Building on Curran’s article “Implementation science made too simple”—which defines “the thing” as the intervention, practice, or innovation in need of implementation support—we offer a typology of four very basic ways to conceptualize policy in implementation science research. We provide examples of studies that have conceptualized policy in these different ways and connect aspects of the typology to established frameworks in the field. The typology simplifies and refines related typologies in the field.

Four very basic ways to think about policy in implementation science research.

1) Policy as something to adopt: an evidence-supported policy proposal is conceptualized as “the thing” and the goal of research is to understand how policymaking processes can be modified to increase adoption, and thus reach, of the evidence-supported policy. Policy-focused dissemination research is well-suited to achieve this goal.

2) Policy as something to implement: a policy, evidence-supported or not, is conceptualized as “the thing” and the goal of research is to generate knowledge about how policy rollout (or policy de-implementation) can be optimized to maximize benefits for population health and health equity. Policy-focused implementation research is well-suited to achieve this goal.

3) Policy as context to understand: an evidence-supported intervention is “the thing” and policies are conceptualized as a fixed determinant of implementation outcomes. The goal of research is to understand the mechanisms through which policies affect implementation of the evidence-supported intervention.

4) Policy as strategy to use: an evidence-supported intervention is “the thing” and policy is conceptualized as a strategy to affect implementation outcomes. The goal of research is to understand, and ideally test, how policy strategies affect implementation outcomes related to the evidence-supported intervention.

**Conclusion:**

Policy can be conceptualized in multiple, non-mutually exclusive ways in implementation science. Clear conceptualizations of these distinctions are important to advancing the field of policy-focused implementation science and promoting the integration of policy into the field more broadly.

Contributions to the literature

- This Debate article aims to provide clear, concise guidance about how policy can be conceptualized in implementation science research.
- A typology of four ways of thinking about policy in the field is presented: (1) policy as something to adopt, (2) policy as something to implement, (3) policy as context to understand, and (4) policy as strategy to use.
- Examples are provided of studies that have conceptualized policy in these different ways.
- The article consolidates and simplifies previously published typologies and frameworks that are relevant to policy-focused implementation science.

## Background

Policy is receiving increasing attention in the field of implementation science [1–12]. Although the use of research evidence in policymaking and implementation have been studied in social science and public administration literatures for over half a century [13], the field of implementation science in health has historically been less attentive to policy [14]. This, however, is changing. Recent reviews have summarized measures of health policy implementation [8, 15, 16], synthesized evidence on policymaker-focused dissemination strategies [9], and identified priorities for methodological innovation in policy implementation research [10]. Calls for the field to place greater emphasis on health equity and structural racism also highlight the importance of studying how social and economic policies, and their implementation, contribute to health inequities [4–7].

Despite growing enthusiasm for policy-focused work in implementation science, conceptualizing policy questions in the field often feels like the proverbial problem of hammering a square peg into a round hole. This is in part because implementation science emerged from the evidence-based medicine movement [17]. As a result, clinical and organizational—as opposed to policy—settings are the implied, if not explicit, focus of core tenets and constructs in the field. In other words, policy-focused research questions often do not neatly fit within prevailing ways of thinking in the field of implementation science.

This concise Debate article aims to help implementation science researchers address this mismatch. Inspired by Geoff Curran’s article “Implementation science made too simple,” [18] the article is intentionally brief and aims to avoid the pitfall of not being easily comprehendible to audiences with little prior knowledge about implementation science research [19, 20]. We offer a typology of four *very basic* ways that policy can conceptualized in implementation science. We hope that our simplified typology will provide clear, concrete, and concise guidance to help implementation scientists conceptualize and conduct policy-focused research. Furthermore, we hope that it will support the integration of policy into implementation science research that is focused on clinical, organizational, and community settings. The guidance offered here simplifies and refines related typologies in the field [10, 11, 21–24].

## Four ways to think about policy in implementation science

**Fig. 1:**
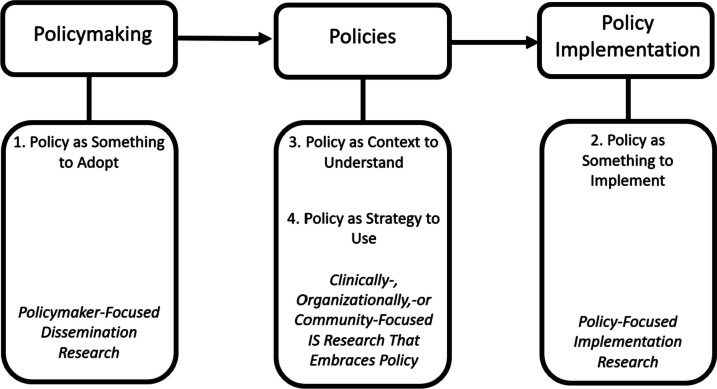
Four ways to conceptualize policy in implementation science

Figure 1 enumerates four basic ways that policy can be conceptualized across three sequential domains: *policymaking*, *policies* (codified in statutes and rules), and *policy implementation*. Across these domains, it is imperative to specify whether the policy of focus is public (i.e., government, also known as “big P policies”) or private (e.g., insurance company, health care system, also known as “little p policies”) [25]. For public policies, it is also important to consider whether the policy is made by elected officials or administrative officials and the level of government where the policy will be/was enacted (e.g., federal, state, or city/county in the case of the USA) [26]. Furthermore, a “policy” can be operationalized as a policy in its entirety (e.g., a bundle of policy provisions within a law), a sub-set of provisions, or an individual provision. Finally, policies change over time. Policy-related research questions can be oriented towards a new policy, a recently modified policy, a longstanding policy, or de-implementation [27] of an ineffective, burdensome, or harmful policy.

### Policy as something to adopt

Here, a specific policy proposal is the focus of the research question and conceptualized as “the thing” per Curran’s terminology [18] (Curran defines “the thing” as the intervention, practice, or innovation which is in need of dissemination or implementation support). The policy should be supported by an existing body of evidence—typically produced from rigorous quasi-experimental studies—indicating that more widespread of adoption of the policy (e.g., across more states or health systems) would be beneficial from a public health and health equity perspective. When conceptualizing policy this way, the goal of research is to understand how policymakers’ minds can be changed so that their behaviors contribute to policymaking processes that increase the adoption, and thus reach, of the evidence-supported policy. These types of studies could also focus on understanding and intervening on the determinants of instrumental use of research evidence (i.e., research evidence directly informing policy decisions) in policymaking [28].

Policy-focused dissemination research is well-suited to achieve this goal. Such research seeks to understand how research evidence can be most effectively packaged and communicated to policymakers and integrated into policymaking processes. Examples of studies that conceptualize policy in this way include audience research to inform how evidence about the policy (or the issues it addresses) is disseminated to policymakers [29–31], survey-based experiments [32–34] and field experiences [35–37] testing the effects of different messages on policymaker engagement with evidence and knowledge and attitudes about the policy or issues it addresses, and models [38] and interventions [39] that aim to improve the use of instrumental research evidence in policymaking. Brownson’s Model of Dissemination Research [25] and Kingdon’s Multiple Streams theory of policymaking [40] are examples of frameworks that have guided implementation science studies that conceptualize policy in this way [41].

### Policy as something to implement

Here, a specific policy is also the focus and conceptualized as “the thing.” The policy of focus does not need to be evidence-supported, however, as policies without an evidence base are frequently implemented in the real world. The extent to which these policies produce benefits or harms to public health, and ameliorate or exacerbate health inequities, can often hinge upon implementation processes. When conceptualizing policy in this way, the goal is to generate knowledge about how the rollout of polices can be optimized to maximize benefits for population health and health equity.

Policy-focused implementation research is well-suited to achieve this goal and has a long history in the field of public administration research [13]. Examples of such research include studies that assess readiness to implement a policy before it is rolled out; describe the process through which a policy was implemented, the extent to which it was enforced, and the actors involved with implementation; identify determinants of implementation outcomes; uncover the mechanisms through which policy implementation processes affect outcomes and their distribution across social groups; and observationally contrast or experimentally test strategies aimed at improving policy implementation outcomes (as well as policy effectiveness outcomes in the case of a hybrid study) [42–48]. Bullock and colleagues’ integrated framework of policy implementation [24] and Lipsky’s theory of Street-level Bureaucracy [49] are examples of frameworks that may support studies that conceptualize policy this way.

### Policy as context to understand

Here, an evidence-supported clinical, organizational, or community intervention is “the thing,” and policy is conceptualized as a fixed determinant of implementation outcomes. The goal, when conceptualizing policy this way, is to understand the mechanisms through which policies affect implementation of the intervention, and how clinically, organizationally, or community-targeted implementation strategies might be selected and tailored for different policy contexts [50]. Although the policies of interest are technically modifiable—they always are because policies are made through social processes—they are conceptualized as fixed here because questions about how to change policies are beyond the scope of the central research question (such questions are primary when conceptualizing policy as in #1 above). Conceptualizing policy as a fixed determinant is consistent with how policy can be thought of as bridging and outer-setting factors in the Exploration, Preparation, Implementation, Sustainment framework [51] and Consolidated Framework for Implementation Research [52, 53]. 

### Policy as strategy to use

Here, an evidence-supported clinical, organizational, or community intervention is “the thing,” and policy (either “big P policy” or “little P policy”) is conceptualized as a strategy to affect implementation outcomes. When conceptualizing policy this way, the goal is to understand, and ideally test, how adopting and amending polices may affect implementation of an intervention. Randomized-controlled designs can be used to answer such questions. However, quasi-experimental or simulation modeling approaches are typically more feasible in which outcomes are compared across geopolitical jurisdictions (e.g., states) or health systems that different policies “on the books” at the same time. Many established typologies of implementation strategies conceptualize policy as a strategy to use. Examples include the Expert Recommendations for Implementing Change compendium (e.g., strategies such as “mandate change,” “change liability laws,” “change accreditation or membership requirements”) [54], “policy categories” in the Behavior Change Wheel (e.g., “legislation, “regulation,” “fiscal measures”) [55], and the Policy Ecology Framework (e.g., “EBP legislation,” “parity laws,” loan forgiveness”) [56].

## Conclusion

Policy can be conceptualized in multiple, non-mutually exclusive ways in implementation science. Clear conceptualizations of these distinctions, we argue, are important to advancing the field of policy-focused implementation science and prompting the integration of policy into the field more broadly. This typology offers four ways to conceptualize policy in implementation science, but there are likely additional ways of thinking about policy in the field. We hope that this simplistic typology will serve as a is a point of departure for more policy-focused intellectual exploration, dialogue, and research in the field of implementation science.

## Data Availability

Not applicable.
